# A quantitative assessment of the contribution of small standing water bodies to the European waterscapes – case of Estonia and France

**DOI:** 10.1016/j.heliyon.2019.e02482

**Published:** 2019-09-24

**Authors:** Jaanus Terasmaa, Pascal Bartout, Agata Marzecova, Laurent Touchart, Egert Vandel, Tiiu Koff, Quentin Choffel, Galina Kapanen, Véronique Maleval, Marko Vainu, Camille Millot, Zoubida Qsair, Mohammad Al Domany

**Affiliations:** a Institute of Ecology, School of Natural Sciences and Health, Tallinn University, Narva mnt 25, Tallinn, 10120, Estonia; b Department of Geography, EA 1210 CEDETE, Orleans University, UFR Collegium LLSH, 10 rue de Tours, BP 46527, 45065, Orléans cedex 2, France; c Department of Geography, Limoges University, Faculté des Lettres et Sciences Humaines, 39E rue Camille Guérin, 87036, Limoges cedex, France

**Keywords:** Aquatic ecology, Ecosystem change, Global ecological change, Nature conservation, Natural resource management, Sustainable development, Physical geography, Remote sensing, Hydrology, Freshwater biodiversity, Lakes, Ponds, Reservoirs, Inventory, Detection limit, Shoreline

## Abstract

The abundance and properties of small standing water bodies (SSWB) is globally not well known for their ecological importance is undervalued and their detection suffers from technical limitations. In the current study, we used a combination of GIS-based methods (satellite, orthophoto, ground validation) to evaluate regional estimates of standing water body (SWB) inventories in two geographically different parts of Europe – France, and Estonia. In our study the SWBs surface area threshold limit was 0.00001 km^2^, exceeding the limits of previous studies (>0.002 km^2^). The total number of SWBs in Estonia is 111 552 (2.5 per km^2^) and in France 598 371 (1.1 per km^2^). Our estimates show that the median size of SWBs in Estonia and France is 0.0003 km^2^ and 0.0007 km^2^ respectively, meaning that most of the SSWBs are not included in the global inventories, and their number is therefore underestimated. SSWBs (area below 0.01 km^2^) form a significant share of the total shoreline length of SWBs, 70.3% in Estonia and 58.8% in France. As nearshore areas are often very productive with diverse habitats, the SSWBs hold a crucial role in maintaining biodiversity. Our results provide quantitative evidence that SSWBs are vital and abundant landscape elements, freshwater resources, and habitats that should not be ignored in global inventories.

## 1 Introduction

All standing water bodies (SWB) are essential for landscape biodiversity. Furthermore, small standing water bodies (SSWB) (area below 0.01 km^2^) have been shown to support more freshwater species as a whole than rivers or big lakes across different countries (Williams et al., 2004; Biggs et al., 2005; Karaus et al., 2005; Demars and Edwards, 2007; Davies et al., 2008; Oertli et al., 2009; Martinez-Sanz et al., 2012; Lischeid et al., 2018). SWBs form networks that can play a significant role in providing five major types of ecosystem services: flood prevention, water storage, nutrient and other pollutant mitigation, carbon sequestration and biodiversity preservation; but also social and cultural benefits including improved recreational possibilities and well-being (Hill et al., 2018). Habets et al. (2018) describes the increasing importance of the cumulative hydrological impact of the small artificial reservoirs, for their number rises as a mitigation measure to adapt with climate change. However, SSWBs are often not connected to a stream network, are shallow and with a high perimeter to volume ratio, leaving them linked only with the surrounding terrestrial environment and land-use practices (Pätzig et al., 2012; Gołdyn et al., 2015). The existence of a high spatial density of aquatic microenvironments in the landscape is an essential factor for survival and migration for a wide variety of species (Smith et al., 2002). Biggs et al. (2017) concluded in their extensive literature review that SSWBs are areas of high biodiversity, especially for macrophytes, amphibians and aquatic micro- and macroinvertebrates. SSWBs have been the numerically dominant freshwater habitats and despite their lower average alpha diversity, at regional level small ponds and lakes have high gamma diversity (Williams et al., 2004). Even less diverse small water bodies (such as bog pools) often support rare, unique plant and animal assemblages and contribute to the diversity of habitats. Hence their contribution to landscape biodiversity cannot be disregarded (Beadle et al., 2015). Collectively, most of the global terrestrial-aquatic interface (perimeter) is in SSWBs (Verpoorter et al., 2014), and this zone harbours some of the most productive habitats on Earth (Wetzel, 1992).

Premke et al. (2016) claims that small and shallow SWBs can act entirely as littoral zones and the whole system can be biogeochemically active. The organic carbon burial rate was found to be significantly high (2000 g m^−2^ yr^−1^) in small impoundments. The world's farm ponds alone may bury four times more organic carbon than the oceans combined or 33% of what the world's rivers deliver to the sea (Downing et al., 2008). Contrastingly, Mendonça et al. (2017) proposed more modest estimations - inland lakes and reservoirs together contribute to carbon burial only as much as the oceans. They also conclude that the small water bodies, mainly small agricultural ponds, have higher rates of OC burial per area unit. The global estimates of inland water OC burial range from 0.2 to 1.6 Pg C per year (references in Mendonça et al., 2017). The estimates vary depending on the types of water bodies included in the studies, but also due to the lack of measurements in different regions and environments. Therefore, the main issue in the quantitative estimates of the ecological, biochemical and hydrological significance of SWBs is the lack of reliable estimates of abundance of SWBs as well as the high spatial and temporal variability of their occurrence.

Rouillard et al. (2017) claims that biodiversity in aquatic systems is declining, and environmental policies have been unable to halt and reverse this trend. Until now, the SSWBs have been disregarded in environmental management and protection policies. For example, the European Water Framework Directive 2000/60/EC proposes the threshold surface area of SWBs for typology and reporting as 0.5 km^2^. At the same time, studies have shown (e.g., Hill et al., 2018), that numerous small habitats support higher taxonomic richness and conservation value than one large habitat and demand fewer resources for protection and restoration. SWBs with a small volume require protection due to their limited ability to dilute and retain pollution, and therefore their ecosystems are highly susceptible to inputs of even small amounts of pollutants.

The European watershed has numerous types of SWBs: natural lakes with different genesis, bog pools (formed in the surface depressions of raised bogs), and man-made reservoirs, farm ponds, fish ponds, etc. Formerly glaciated landscapes in Europe are rich with SSWBs called “kettle holes” (or “potholes” in North America) (Lischeid et al., 2018). Definition of the different types of water bodies (e.g., pond versus lake) tends to be country-specific with no universal definition of the boundary size or genesis to distinguish lakes and ponds. The term “pond” is often used in a more general sense and could describe both natural and artificial SWBs (Pätzig et al., 2012), or as in Estonia, strictly meaning only artificial SSWBs. Different authors have set various lower and upper limits for the surface area of ponds. Lower limits range from 0.000001 km^2^ to 0.001 km^2^ (Biggs et al., 2005; Oertli, 2013; Bartout, 2015), upper limits from 0.01 km^2^ to 2 km^2^ (Jedicke, 1989; Céréghino et al., 2008; Oertli et al., 2009).

The availability of information about the distribution of SWBs varies across Europe. SSWBs can be well represented in the state-level inventories and studies (e.g., <0.1 km^2^ in Meybeck (1995), <0.01 km^2^ in Ryanzhin (2005), or <0.0005 km^2^ in Kuusisto and Raatikainen (1988)). In most cases, the SWBs in smaller size ranges remain undocumented. Recent research (Ecrins Lake Database) carried out by the European Environment Agency confirms the systematic underestimation of SWBs in European and local water policies. According to the analysis of the OpenStreetMap (OSM) database by Bartout et al. (2015), the total number of SWBs in all EU countries is ca. 1 million. At the same time, they concluded that to achieve genuinely reliable estimates, each geographical region requires a specific function adapted to its hydrological characteristics. Compared to national inventories this number covers on average over 80% of SWBs in the size class >0.1 km^2^. Moreover, the models for global inventories so far are not designed for estimating the relative abundance of SSWBs (detection limit is 0.002 km^2^ by Verpoorter et al. (2014) and 0.005 km^2^ by Feng et al. (2016)) and provide different estimates. Detection limit using LIDAR-based methodologies (Lang and McCarty, 2009) is similar (∼0.002 km^2^ in Toscano et al. (2014)) or even lower (0.008 km^2^ according to the USGS (Heidemann, 2012)).

In this paper, we aim to:

(1) introduce a bottom-up GIS-based approach for estimating the number of SWBs combining state inventories with orthophotos and ground-validated data;
(2) evaluate the abundance and size distribution of SWBs in two geographically and historically different countries, Estonia and France;
(3) assess the role of SSWBs in connection with biodiversity and landscape connectivity.

Estonia and France were selected as study sites for the following reasons:

- both countries have well studied SWB inventories that allow to describe and analyse the distribution of SSWBs reliably.
- to test the hypothesis that regardless of the formation and history of SWBs, the relative abundance and importance of SSWB in total inventory is similar in different regions.

Estonia is rich in natural SWBs while artificial SWBs dominate the waterscape of France. We use these inventories to discuss the advantages and limits of the applied quantitative approach of SSWBs in the broader historical and methodological context to outline the future directions in the research of SWBs in European waterscapes.

## 2 Study areas

### 2.1 Estonia

**Fig. 1:**
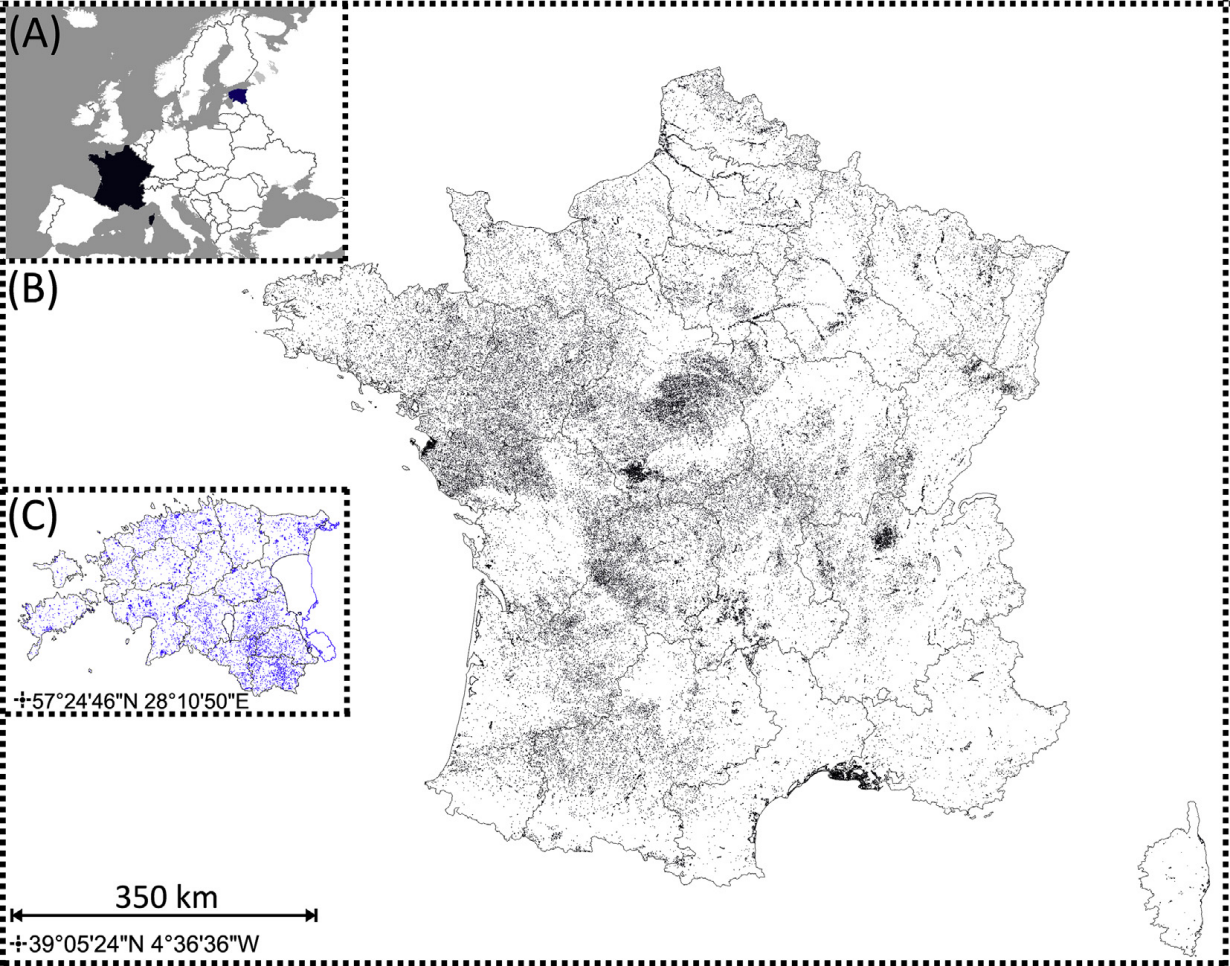
Locations of the study areas (A). Distribution of standing water bodies in France (B) (Estimates by Bartout and Touchart (2013) based on a combination of the French database IGN BD Topo) and Estonia (C) (Estonian Topographic Database).

The territory of Estonia (45 215 km^2^) is situated on the shore of the Baltic Sea and belongs to the European Boreal region (Fig. 1). As a part of the East-European Plain, Estonia is characterised by a flat surface topography: over 60% of the country's territory lies at an absolute height of 0–50 m (Raukas and Teedumäe, 1997). The majority of Estonian lake basins formed during the retreat of the continental ice. Lakes are rather shallow, ca 75% of them have a depth less than 10 m (Ott and Kõiv, 1999). In Estonia the wetlands are common landscape features, covering 20% of the territory. Peat-rich mires with characteristic bog pools are abundant (Ilomets and Kallas, 1995). Creation of artificial SWBs, mainly to ensure operation of water mills or fish breeding, has been relatively common in Estonia throughout the centuries. New artificial water bodies have been constructed lately to diversify landscapes and create swimming possibilities or as an outcome of sand and gravel excavations.

### 2.2 France

The territory of France in Europe covers 547 030 km^2^ (Fig. 1). France has a variety of landscapes, from mountain ranges of the Alps in the south-east to coastal plains in the north and west, the Massif Central in the south-central and Pyrenees in the south-west. Except for the few mountain margins, France was not glaciated during the Last Glacial Maximum (Fort and André, 2014). The SWBs can be roughly distinguished into two different categories: natural water bodies (mountain and coastal lakes) and man-made water bodies situated in floodplains, lower elevations, and plateaus. Most of the French SWBs are artificial (∼98%). The practice of creating fishponds was introduced in the Middle Ages and had multiple agricultural or industrial functions (Bernard, 2008). At present, ponds built since 1960 have mainly recreational purposes such as fishing and swimming.

## 3 Materials and methods

This study utilises two sets of national level (country-specific) water-body inventories. The water bodies were divided between six size classes according to their area (<0.0001 km^2^, 0.0001–0.001 km^2^, 0.001–0.01 km^2^, 0.01–0.1 km^2^, 0.1–1.0 km^2^ and >1.0 km^2^). For SWBs of both countries, a series of parameters (total count, area, perimeter, density, etc.) were calculated for each of the size classes. These parameters were also specifically assessed for SWBs with size below 0.002 km^2^ to estimate the typical cut off by the detection limit applied in the most recent global inventory based on GLOWABO data from satellite imagery (Verpoorter et al., 2014). The analyses were performed using ESRI ArcGIS 10.2.2. software.

Estonian Topographic Database (ETD) by the Estonian Environmental Agency is based on the topographic and orthophoto maps (Estonian Land Board (geoportaal.maaamet.ee)). The ETD contains over 111 000 SWBs placing each in one of the following categories: lakes, reservoirs, artificial lakes, bog pools and ponds. The current minimum mapping size for SWBs is 0.00002 km^2^ (and 0.0001 km^2^ for bog pools), but it also contains smaller objects. The ETD is continuously updated, and uncategorised SWBs are moved to correct categories. In this paper, we use the 2017 version of ETD. Lake Peipsi was excluded from analyses because of its large size (one order of magnitude higher than the second largest SWB in Estonia) and because it is a transboundary waterbody (shared with Russia).

For quality check, we visually verified the Estonian dataset by running a random validation of the ETD against orthophotos from Estonian Land Board. A hundred of 1 × 1 km plots were randomly selected from the area covering the whole landmass of Estonia. The amount of SWBs in ETD in these plots was visually compared with the latest orthophotos from 2012–2016 (pixel size 25–50 cm); if unavailable, the most recent available orthophotos were used. When Estonian Land Board orthophotos were not clear enough, satellite photos from Google (maps.google.com) were used for comparison.

The French inventory (Bartout and Touchart, 2013) contains more than 550 000 objects bigger than 0.0001 km^2^. The database was compiled by combining three sources: orthophotos (with 10 m pixel resolution), cartographic maps (1: 500 to 1: 25 000 scale) and ground validation. Specifically, in the Limousin region, 15 000 SWBs in all size classes were revised *in situ* as described in detail in Bartout (2006)*.* The objects smaller than 0.0001 km^2^ were extracted from *OpenStreetMap* database, but their number is still underestimated (Bartout and Touchart, 2016). The quality checks of the French SWB database have been described in several papers (Bartout, 2006; Bernard, 2008; Bartout et al., 2015).

## 4 Results

### 4.1 Quality of data

**Fig. 2:**
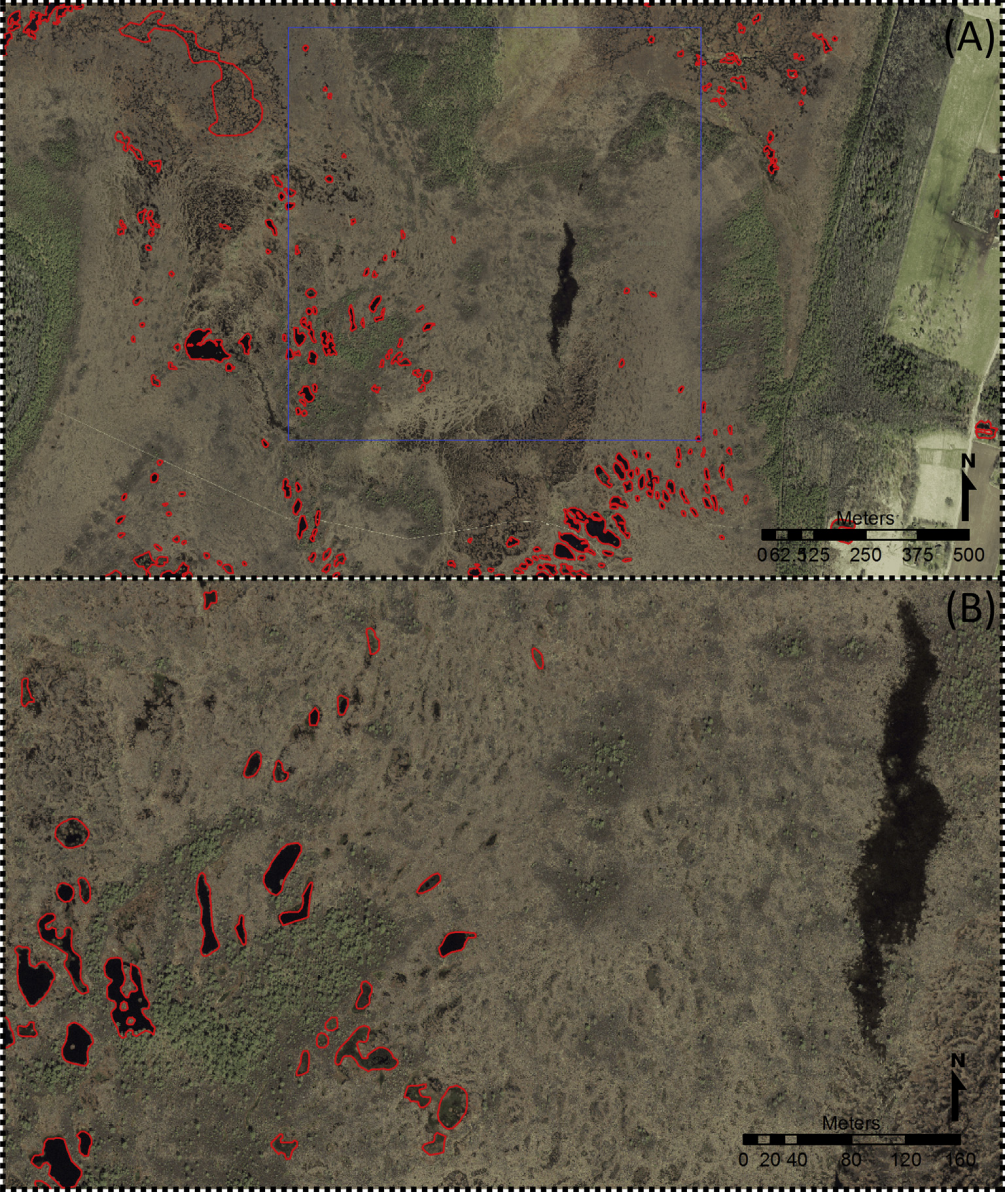
Discrepancies in bog pool data in the Nigula Bog (Estonia). (A) The blue square is one of the randomly selected 1 × 1 km plots. Red contours are SWBs from Estonian Topographic Database (ETD). (B) Several bog pools visible in the Estonian Land Board orthophoto are missing from the database, whereas some bog pools marked in ETD have almost disappeared.

Among the hundred plots analysed for quality check the ETD and orthophotos contained 213 and 245 SWBs, respectively (difference 15.0%) (Table 1). The most significant inaccuracy in the Estonian inventory is in the bog pools category (Fig. 2). Excluding the bog pool category, ETD and orthophotos included 119 and 113 SWBs, respectively (difference 5%). In summary, the ETD underestimates the number of SWBs in the bog pool category and slightly overestimates the number of SWBs in the other categories (at least partly due to the human transformation of the landscape). In general ETD is suitable for fulfilling the aims of this research.

**Table 1:** Results of the quality check (100 1 × 1 km plots) based on the comparison between the Estonian Topographic Database and orthophotos.

	ETD	Orthophoto	Difference, %
Number of plots with SWBs	45	47	
All detected SWBs	213	245	15.0
bog pools	94	132	40.4
all other SWBs	119	113	5.0
Average number of SWBs per km^2^	4.73	5.21

The quality checks of the French inventory have shown that the cartographic maps IGN 1:25 000 contained 64% of SWBs featured in the inventory. On the national level, the usage of orthophotos gave the best results. In the Limousin region, 94.6% of water bodies in all size categories were detected. In Vauvre basin (Central France), 98.6% of all SWBs were in the database. Due to the high number of very small SWBs (less than 0.0001 km^2^) in the Sologne region, the detection rate was lowest (88.5%). In this region SSWBs (dew-ponds, “mares” in French) are abundant, but as historically they were not subjects of the declaration, they have not been included on maps or in databases.

### 4.2 Abundance, size distribution and perimeter of SWBs in Estonia and France

**Fig. 3:**
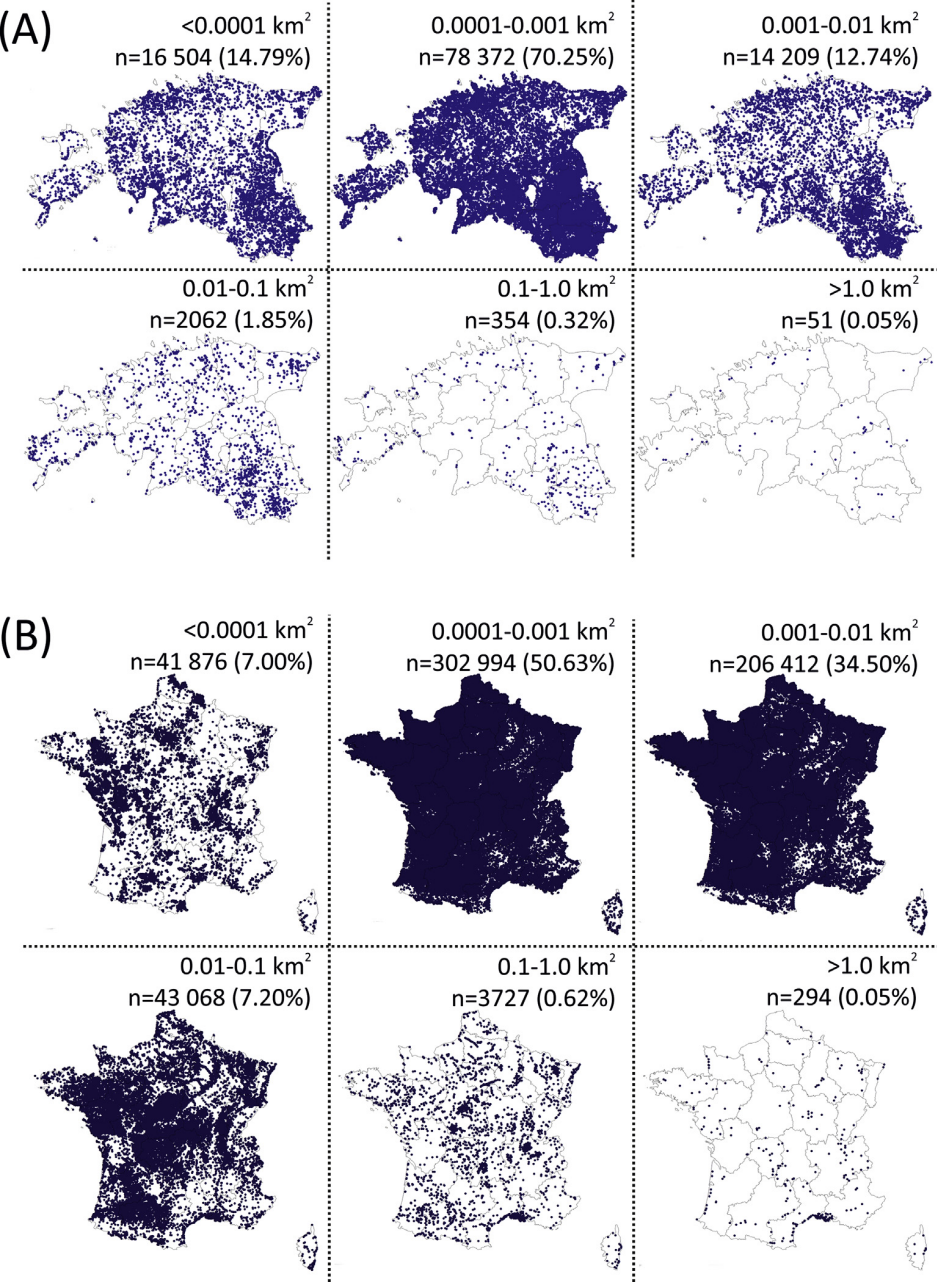
SWBs abundance in different size classes. (A) Estonia and (B) France. Every dot on the map represents a single SWB. The numbers in the corners indicate total counts of SWBs in size classes and their share in percentages of the total SWB counts.

The abundance of SWBs in different size classes are shown in Fig. 3. Estonian database contains 111 552 and France database 598 371 SWBs. Both in Estonia and France, the majority (70.25% and 50.63%, respectively) of SWBs were detected in the size class of 0.0001–0.001 km^2^. Altogether 97.78% of the SWBs in Estonia and 92.13% in France are smaller than 0.01 km^2^. In both countries, SWBs in the size class bigger than 1 km^2^ have the lowest share – only 0.05% of the total number of SWBs.

**Fig. 4:**
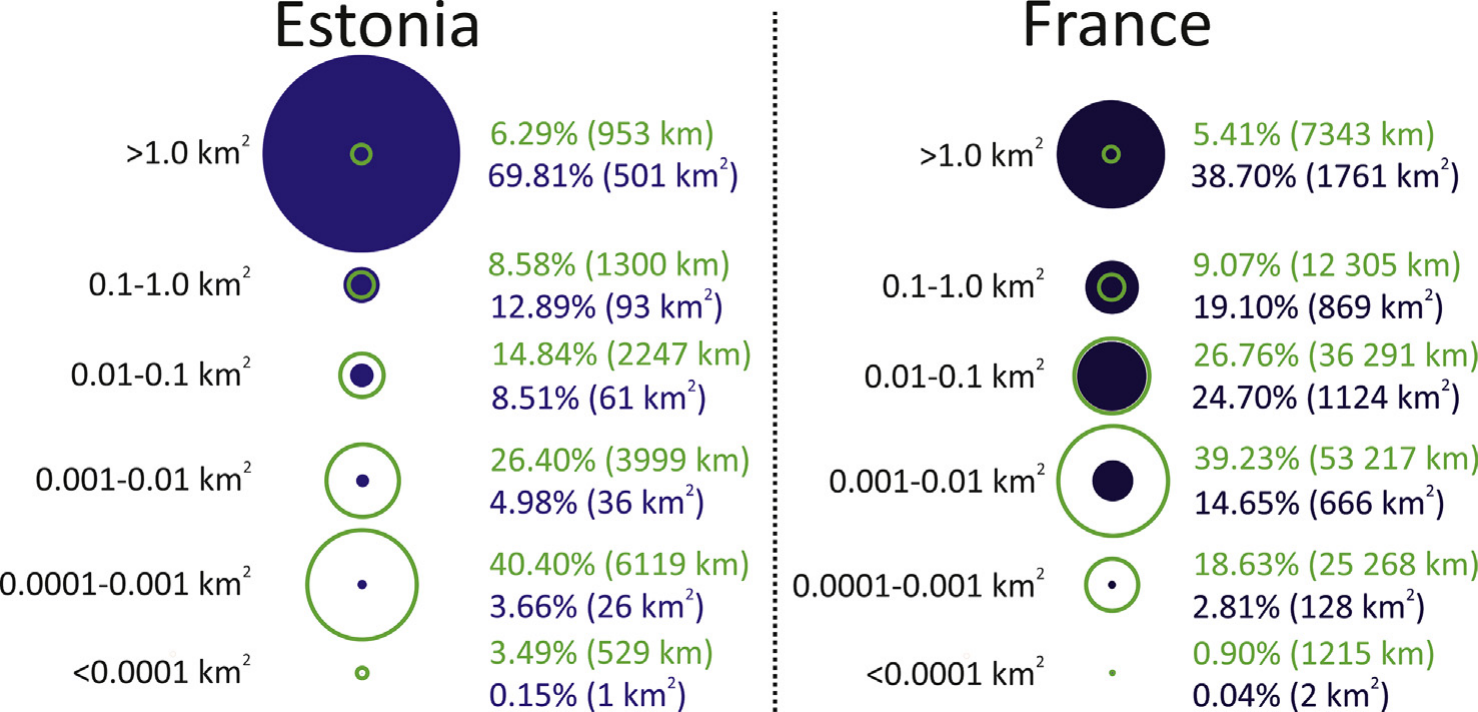
The comparison of size classes of SWBs in Estonian Topographic Database (ETD) and French national inventory. The green colour indicates the calculated total shoreline length (%) and the blue colour indicates the total area (%). Lake Peipsi is excluded (area 3541 km^2^, shoreline length 814 km) from Estonian dataset.

The distribution of the total perimeter and the total area between the size classes is demonstrated in Fig. 4. In Estonia, SWBs smaller than 0.01 km^2^ form only 8.79% from the total area, but they cover 70.29% of the total perimeter of SWBs. In France, SWBs under 0.01 km^2^ make up 17.50% of the total area and 58.76% of the total perimeter of SWBs. In France, the highest share of the perimeter is in the size class of 0.001–0.01 km^2^ (median perimeter 0.11 km), and in Estonia, within the size range of 0.0001–0.001 km^2^ (median perimeter 0.07 km).

Summarised statistics of the national-level inventories from Estonia and France are given in Table 2. Many parameters are similar for both countries, even though the number of SWBs is five times higher in France and the origin of the water bodies is very different. As Lake Peipsi is excluded from the analysis, the area of the biggest SWB is similar in both countries. The same applies to the mean area of SWBs. In France, the majority (98%) of SWBs are man-made. In Estonia, 52% of the SWBs are man-made, and 48% are natural SWBs. Whereas 1 559 of the natural SWBs are lakes, and 45 309 are bog pools.

**Table 2:** Parameters of the national-level SWB inventories.

	Estonia		France	
No. of SWBs	111 552		598 371	
The proportion of the man-made SWBs (%)	52		98	
SWB density per km^2^	2.5		1.1	
	Perimeter, km	Area, km^2^	Perimeter, km	Area, km^2^
Minimum	0.013	0.00001	0.008	0.00001
Maximum	207	269	267	241
Median	0.069	0.00027	0.114	0.00072
Mean	0.136	0.0064	0.227	0.0076
Sum	15 147	718	135 639	4550

Evaluation of the Estonian and French databases against the detection limit used in the GLOWABO database showed that most of the SWBs are smaller than 0.002 km^2^ (Table 3), respectively 92,3% and 71.6%.

**Table 3:** SWBs below the detection limit (0.002 km^2^) of the GLOWABO database.

	Estonia	France
<0.002 km^2^ SWB count, %	92.3	71.6
<0.002 km^2^ SWB total area, %	5.4	5.5
<0.002 km^2^ SWB total perimeter, %	54.7	30.1

## 5 Discussion

The abundance and size distribution of SWBs in Estonia and France shows the quantitative importance of small natural and man-made standing water bodies. The median size of SWBs in Estonia and France is smaller (0.0003 km^2^ and 0.0007 km^2^, respectively) than the size limit (0.002 km^2^) in the GLOWABO database (Verpoorter et al., 2014) or in LIDAR based approaches (Heidemann, 2012; Toscano et al., 2014).

Verpoorter et al. (2014) conclude, based on GLOWABO, that medium and large lakes dominate the global areal extent of lakes, which is in accordance with our results. The importance of SSWBs derives from their total perimeter. In the case of Estonia and France – located in very different geological and historical settings – our results showed that waterbodies with areas below the current remote sensing detection limit of 0.002 km^2^ contribute most to the land-water interface.

### 5.1 The significance of SSWBs for biodiversity

The main argument for cutting off SSWBs from quantitative estimates is their negligible area. However, as our results attest, these water bodies with small areas contribute disproportionately to the nearshore area. The calculated shoreline length (perimeter) of SWBs in Estonia and France shows that most of the terrestrial land-water interface lays in the small (below 0.01 km^2^) SWBs (Fig. 4). This result is consistent with the hypothesis by Wetzel (1992) and renewed by Verpoorter et al. (2014) claiming that the majority of the global terrestrial land-water interface is in SSWBs. The importance of the near-shore and littoral zones derives from the fact that they often represent one of the richest habitats where the primary exchange of matter and energy takes place (Wetzel, 1992).

For example, Davies et al. (2008) found that in the regional level small ponds are the most species-rich aquatic habitats for both wetland plants and macroinvertebrates and have the highest index of species rarity. SSWBs can be considered as the stepping stones for the migration, dispersal and genetic exchange of wild species. Several recent studies have stressed the importance of hydrological connectivity of ponds on general biodiversity. It has been shown that connectivity between ponds increases the species richness of macrophytes (Akasaka and Takamura, 2012), fish (Uchida and Inoue, 2010), phytoplankton (Naselli-Flores et al., 2016) and amphibians (Ribeiro et al., 2011). Although individual SSWBs have lower average alpha diversity than large water bodies, at a regional scale they typically have high gamma diversity (Williams et al., 2004). However, there is also a risk for biotic homogenisation and the consequent loss of beta and gamma diversity because of the migration of non-native species. It should be noted that the total perimeter of SWBs is not a suitable measure of ecological importance for migration. However, due to their long total perimeter small natural and man-made water bodies have to be included in further studies to assess their abundance and their potential contribution to biodiversity.

SSWBs are important for biodiversity on catchment as well as on landscape level – individually they may not be species-rich, but they are abundant and varied and affected by land-use practices in the catchment (Lischeid et al., 2018). There is a strong need for developing the methods to assess the ecological quality of SSWBs based on their specific typology and habitat functions (Pätzig et al., 2012). Collectively they represent a significant proportion of the surface water resource and are critical habitat for threatened freshwater biodiversity, but also play an essential role in supporting higher terrestrial richness and abundance of terrestrial species (Hill et al., 2018).

### 5.2 Bog pools: mapping the vital landscape element

The ETD contains 45 309 bog pools (SSWBs formed in peat bogs), comprising a significant proportion of SWBs, especially in the smallest size categories. However, as our analysis indicated, there are problems with the quantification of bog pools, and they are underrepresented in the Estonian database (Table 1). The reasons for that could be plentiful. Detecting them from orthophotos is cumbersome. Also, most of them are located in remote terrains with limited accessibility and are difficult to verify by ground validation. They can be tiny (median is 0.0002 km^2^) and disappear due to natural processes faster than waterbodies in the mineral ground. The disappearance of bog pools is often also related to the drainage of the wetlands (massively started in the 19^th^ century and expanded in the 20^th^ century) to get more suitable land for forestry or agricultural purposes. The current bog pools are remnants of former abundance. The high margin of error in the estimates of the bog pool number could be considered as a reason to exclude this ecosystem type from the overall country estimates. However, due to the ecological and landscape significance of bog pools, we retained them in the estimate. It is essential to understand their spatial distribution and abundance because of their importance for the landscape biodiversity and species connectivity. Due to low pH and nutrient levels, bog pools can be considered as relatively species-poor ecosystems. However, they significantly increase the habitat diversity, and they house important communities of macroinvertebrates, as well as rare and endangered species (Beadle et al., 2015). For example, several studies demonstrate bog pools to be suitable habitats for rare and endangered odonates such as *Aeshna caerulea*, *Somatochlora arctica*, *Leucorrhinia dubia* in Scotland (Maitland, 1999) and critically endangered *Coenagrion hastulatum* in Estonia (van Kleef et al., 2012). The latter study also identified six species of vulnerable, threatened, or critically endangered Trichoptera. Bog pools are (e.g. in Finland, Sweden, Estonia) or have been (e.g. in Netherland, Germany) wide-spread in various regions of Europe. Therefore the understanding of the abundance and development of bog pools is vital from the perspective of the European waterscape.

### 5.3 Quantitative and qualitative importance of man-made SWBs

Man-made water bodies comprise a significant proportion of the small size classes of SWBs in France as well as in Estonia (Table 2). Majority of SWBs in France are artificial (Table 2), and many of them are under the protection as habitats for protected species (Bartout et al., 2015). The ETD demonstrates the quantitative significance of small man-made SWBs also in Estonia, where the overall number of man-made waterbodies slightly exceeds the natural ones. The natural waterbodies still form the majority of the total area because most of the man-made waterbodies are under 0.01 km^2^ and only 8 have an area over 1 km^2^. Natural water bodies have a higher share of the perimeter in the size classes above 0.1 km^2^. In Estonia over 3000 of man-made SWBs are in protected areas, and protected species inhabit over 2000 man-made SWBs outside of the protected areas.

These findings are in line with recent studies that emphasise the importance of man-made SWBs for the conservation of biodiversity (Céréghino et al., 2008; Pätzig et al., 2012; Hill et al., 2018). For example ponds, despite their small size, contribute to the regional diversity disproportionately when compared to streams, rivers or large lakes (Oertli et al., 2009; Williams et al., 2010). In many cities, ponds are constructed to treat and store stormwater runoff or stabilisation ponds to treat wastewater.

Our study provides a snapshot of the current situation; however, to understand the role of human practices it is essential to put it into historical context. Regarding temporal variability, over the last century, many countries have experienced a considerable net loss of SWBs due to the land-use change (Williams et al., 2010). The estimates from Great Britain suggest that during the last 120 years the number of ponds has been reduced from 800 000 to around 400 000 (Biggs et al., 2005). However, in France, the trend is different – the overall number of small ponds has significantly increased since the 1950s (Bartout et al., 2015). Creating ponds in urban settings and in farmed landscapes to collect, store and to trap sediment/pollution runoff before gradual release into water courses is one of the nature-based solutions suggested for mitigating the anthropogenic impact (Sutherland et al., 2014).

## 6 Conclusions

The number and properties of SSWBs are globally not well known for their importance is generally undervalued or their detection suffers from technical limitations. While big lakes and reservoirs are extensively studied and protected, SSWBs collectively represent a significant proportion of the surface water resource and are critical habitat for threatened freshwater species.

In the current study, we used a combination of GIS-based methods (satellite, orthophoto, ground validation) to improve the reliability of estimates of standing water bodies in Estonia and France. Mapping of SSWBs has often been a complicated task not only because of their small size but also due to their temporal dynamics, for example, the creation of man-made ponds or loss of bog-pools due to drainage of the surrounding landscape.

The total number of SWBs in Estonia is 111 552 (2.5 per km^2^) and in France 598 371 (1.1 per km^2^). Our estimates show that the median size of SWBs in Estonia and France is 0.0003 km^2^ and 0.0007 km^2^, respectively. These results suggest that most of the SWBs in Europe are smaller than 0.002 km^2^, which is the detection limit of the global inventories (using satellite imagery or LIDAR-data). Therefore, we can infer that most of the SSWBs are not included in the global inventories.

The calculated shoreline length of SWBs shows that of the terrestrial land-water interface 70.3% in Estonia and 58.8% in France is in small (below 0.01 km^2^) SWBs and only ∼6% in SWBs bigger than 1 km^2^. Nearshore areas are known to contain very productive and diverse habitats. Hence, the SSWBs hold a crucial role in the hydrological connectivity and general biodiversity, and they should not be neglected while inventorying SWBs.

## Declarations

### Author contribution statement

Jaanus Terasmaa, Pascal Bartout, Agata Marzecova, Laurent Touchart, Egert Vandel, Tiiu Koff, Quentin Choffel, Galina Kapanen, Véronique Maleval, Marko Vainu, Camille Millot, Zoubida Qsair, Mohammad Al Domany: Conceived and designed the experiments; Analyzed and interpreted the data; Contributed reagents, materials, analysis tools or data; Wrote the paper.

### Funding statement

This study was a part of the Hubert Curien Parrot programme (French Ministry of Research and Education in partnership with the Estonian Research Council, the French Foreign Ministry and the Ministry of Higher Education and Research) project AU5-9.1/7 and was partly supported by Estonian Research Council project IUT18-9 “ENCHANTED”. The authors also acknowledge support from the ERASMUS + program.

### Competing interest statement

The authors declare no conflict of interest.

### Additional information

No additional information is available for this paper.
